# Pharmacological and pharmacokinetic properties of JNJ-40411813, a positive allosteric modulator of the mGlu2 receptor

**DOI:** 10.1002/prp2.96

**Published:** 2014-12-09

**Authors:** Hilde Lavreysen, Abdellah Ahnaou, Wilhelmus Drinkenburg, Xavier Langlois, Claire Mackie, Stefan Pype, Robert Lütjens, Emmanuel Le Poul, Andrés A Trabanco, José María Cid Nuñez

**Affiliations:** 1 Janssen Research & Development, Janssen Pharmaceutica NVBeerse, Belgium; 2 Addex TherapeuticsGeneva, Switzerland; 3 Janssen Research & Development, Janssen-CilagToledo, Spain

**Keywords:** In vitro, JNJ-40411813, mGlu2 PAM, occupancy, pharmacokinetics, sleep–wake EEG

## Abstract

Compounds modulating metabotropic glutamate type 2 (mGlu2) receptor activity may have therapeutic benefits in treating psychiatric disorders like schizophrenia and anxiety. The pharmacological and pharmacokinetic properties of a novel mGlu2 receptor-positive allosteric modulator (PAM), 1-butyl-3-chloro-4-(4-phenyl-1-piperidinyl)-2(1*H*)-pyridinone (JNJ-40411813/ADX71149) are described here. JNJ-40411813 acts as a PAM at the cloned mGlu2 receptor: EC_50_ = 147 ± 42 nmol/L in a [^35^S]GTP*γ*S binding assay with human metabotropic glutamate type 2 (hmGlu2) CHO cells and EC_50_ = 64 ± 29 nmol/L in a Ca^2+^ mobilization assay with hmGlu2 G_*α*16_ cotransfected HEK293 cells. [^35^S]GTP*γ*S autoradiography on rat brain slices confirmed PAM activity of JNJ-40411813 on native mGlu2 receptor. JNJ-40411813 displaced [^3^H]JNJ-40068782 and [^3^H]JNJ-46281222 (mGlu2 receptor PAMs), while it failed to displace [^3^H]LY341495 (a competitive mGlu2/3 receptor antagonist). In rats, JNJ-40411813 showed ex vivo mGlu2 receptor occupancy using [^3^H]JNJ-46281222 with ED_50_ of 16 mg/kg (p.o.). PK-PD modeling using the same radioligand resulted in an EC_50_ of 1032 ng/mL. While JNJ-40411813 demonstrated moderate affinity for human 5HT_2A_ receptor in vitro (*K*_b_ = 1.1 *μ*mol/L), higher than expected 5HT_2A_ occupancy was observed in vivo (in rats, ED_50_ = 17 mg/kg p.o.) due to a metabolite. JNJ-40411813 dose dependently suppressed REM sleep (LAD, 3 mg/kg p.o.), and promoted and consolidated deep sleep. In fed rats, JNJ-40411813 (10 mg/kg p.o.) was rapidly absorbed (*C*_max_ 938 ng/mL at 0.5 h) with an absolute oral bioavailability of 31%. Collectively, our data show that JNJ-40411813 is an interesting candidate to explore the therapeutic potential of mGlu2 PAMs, in in vivo rodents experiments as well as in clinical studies.

## Introduction

Among the metabotropic glutamate (mGlu) receptors, mGlu2, an inhibitory presynaptic autoreceptor, has emerged as a novel therapeutic target for the treatment of psychiatric disorders including schizophrenia, depression and anxiety, which are characterized by glutamatergic dysfunction (Marek et al. 2010; Riaza Bermudo-Soriano et al. 2012; Sanacora et al. 2012).

Testing of selective mGlu2/3 receptor agonists in animal studies involving *N*-methyl-d-aspartate (NMDA) receptor antagonists like phencyclidine (PCP) provided early evidence that mGlu2/3 receptors may represent a novel target for schizophrenia treatment. Both LY354740 and LY379268, potent orthosteric mGlu2/3 agonists, inhibit PCP-evoked increases in glutamate levels and PCP-induced hyperlocomotion in rats (Moghaddam and Adams 1998; Cartmell et al. 1999). A prodrug of another mGlu2/3 receptor agonist, LY404039, improved positive and negative symptoms and was well tolerated in patients with schizophrenia (Patil et al. 2007; Mezler et al. 2010). However, the improvements in schizophrenia-related symptoms were not confirmed in subsequent follow-up trials (Kinon et al. 2011; Stauffer et al. 2013), and hence it is questioned whether only particular symptoms or disease stages exhibit a glutamatergic-based origin (Goff and Coyle, 2001; Marsman et al., 2013), and whether earlier results can be generalized to the broader population of patients with schizophrenia or whether these are specific only to subpopulations of patients.

Activation of mGlu2/3 receptors also results in anxiolytic effects in preclinical models (Rorick-Kehn et al. 2007). In healthy volunteers, LY354740 ameliorated fear-potentiated startle and panic induction after CO_2_ challenge (Schoepp et al. 2003). LY544344, the prodrug of LY354740, showed efficacy in the treatment of patients with generalized anxiety disorder (Dunayevich et al. 2008).

It has been suggested that mGlu2 but not mGlu3 receptor mediates the actions of the mGlu2/3 receptor orthosteric agonists LY379268 and LY404039 in mouse models predictive of antipsychotic activity (Fell et al. 2008). All agonists identified so far, however, lack mGlu2 receptor subtype selectivity and also act on the mGlu3 receptor (Fell et al. 2008; Woolley et al. 2008). Treatment with mGlu2/3 receptor agonists may also have potential limitations in terms of tolerance development (Galici et al. 2005). mGlu2-positive allosteric modulators (PAMs) are therefore developed as an alternative, as they increase the endogenous mGlu2 receptor signaling, have greater selectivity than orthosteric agonists, and may maintain activity based on local, transient, and temporal release of glutamate, possibly reducing the risk of tolerance. Preclinical proof-of-concept studies evaluating the mGlu2 receptor PAMs LY487379 and BINA in models predictive of anxiolytic or antipsychotic activity have demonstrated that PAMs can mimic the effects of direct agonists (Johnson et al., 2003, 2005; Galici et al. 2005, 2006). The structurally novel allosteric potentiator THIIC (also known as LY2607540) also showed activity in models predicting anxiolytic or antidepressant effects (Fell et al. 2011).

Recently, the first clinical data with mGlu2 receptor PAMs were disclosed: AZD8529, tested as monotherapy in a phase 2 schizophrenia trial, was not effective, while the active comparator, risperidone, demonstrated activity (Litman et al. 2014). In an exploratory phase 2a study in schizophrenia, the mGlu2 PAM JNJ-40411813/ADX71149 (1-butyl-3-chloro-4-(4-phenyl-1-piperidinyl)-2(1*H*)-pyridinone) met the primary objectives of safety and tolerability. Moreover, patients treated with antipsychotics who experienced residual negative symptoms were identified as a subgroup of patients who may benefit from add-on treatment with JNJ-40411813 (De Boer et al. 2013; Kent et al. 2013). In the adjunctive treatment of major depressive disorder with significant anxious features, administration of JNJ-40411813 in the dose range tested did not appear to have a clinically significant impact on symptoms (Kent et al. 2014). The clinical results obtained with JNJ-40411813 may greatly increase our understanding of the potential of drugs modulating the mGlu2 receptor and further clinical studies, including in additional indications, are awaited.

Here, we describe the pharmacological and pharmacokinetic (PK) properties of JNJ-40411813, including receptor occupancy and effects on sleep–wake architecture in rats. The activities of JNJ-40411813 in behavioral in vivo models predictive of antipsychotic efficacy are described elsewhere (Lavreysen et al. submitted[Bibr b16]).

## Material and Methods

### Materials

[^35^S]GTP*γ*S (specific activity 1250 Ci/mmol; guanosine 5′-[*γ*-thio]triphosphate) was obtained from PerkinElmer (Boston, MA). Fluo-4-AM and FLIPR Ca^2+^ assay kits were procured from Molecular Devices (Sunnyvale, CA); [^3^H]JNJ-40068782, [^3^H]JNJ-46281222, and [^3^H]MDL-100907 were synthesized at Janssen Research and Development (Beerse, Belgium); [^3^H]DCG-IV and [^3^H]LY341495 were obtained from American Radiolabeled Chemicals Inc. (St. Louis, MO). Saponin was purchased from Calbiochem (San Diego, CA). Glutamate was procured from Sigma-Aldrich (St-Louis, MO). All other reagents were obtained from Merck (Darmstadt, Germany).

**Figure 1:**
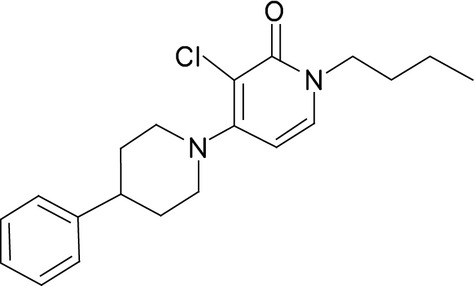
Chemical structure of JNJ-40411813.

JNJ-40411813 (Fig.1) was synthesized at Janssen Research & Development (Toledo, Spain). For in vitro studies, JNJ-40411813 solution was prepared in 100% dimethyl sulphoxide (DMSO) followed by dilution in assay buffer; final concentrations of 1% DMSO were reached for all in vitro assays, except for human mGlu7 and human mGlu8 receptor assays, for which 0.5% DMSO was used. For in vivo studies, JNJ-40411813 (free base) was formulated in 20% hydroxypropyl-*β*-cyclodextrin (HP-*β*-CD) containing 1 equivalent of HCl and the pH was adjusted depending on the dosing route (subcutaneous [s.c.], per oral [p.o.], or intravenous [i.v.]).

### Animals

Male Wistar rats weighing 150 gm and male Sprague–Dawley rats weighing 200 gm were obtained from Charles River Breeding Laboratories (Germany). Male wild-type C57BL/6 and mGlu2 knockout mice were obtained from Deltagen Inc (San Mateo, CA). Animals were housed under standard laboratory conditions (21 ± 2°C; 45–65% relative humidity; and 12/12 h light–dark cycle). The local Ethical Committee in compliance with the Declaration of Helsinki approved all studies.

### In vitro pharmacology

#### Functional mGlu2 assays

[^35^S]GTP*γ*S binding was performed using membranes from Chinese hamster ovary (CHO) cells expressing the rat or human mGlu2 (hmGlu2) receptor. Ca^2+^ assays were performed in G_*α*16_-HEK 293 cells expressing the hmGlu2 receptor. The procedures for both these assays are described in Lavreysen et al. 2013.

#### [^35^S]GTPγS binding to rat brain sections

Autoradiography of agonist-stimulated [^35^S]GTP*γ*S binding in rat brain sections was performed as previously described (Lavreysen et al. 2013). The mGlu2 specificity of the assay was assessed by measuring [^35^S]GTP*γ*S binding in wild-type and mGlu2 knockout mice brain sections in parallel.

#### Radioligand binding to hmGlu2 CHO membranes or rat cortical membranes

Membrane preparation from hmGlu2 receptor stably transfected CHO cells and rat cortex as well as [^3^H] LY341495, [^3^H]JNJ-40068782, and [^3^H]DCG-IV binding experiments were performed as in Lavreysen et al. (2013). For [^3^H]LY341495 competition binding assays, the data were calculated as percentage of total binding measured in the absence and presence of JNJ-40411813. For [^3^H] DCG-IV, saturation binding curves plotting specific binding versus the amount of free radioligand were analyzed using GraphPad Prism (San Diego, CA) (version 4.02, nonlinear regression, one site binding).

Displacement studies, on both membranes prepared from hmGlu2-expressing CHO cells as well as rat cortex tissue, were performed using 4 nmol/L of [^3^H]JNJ-46281222 (a specific mGlu2 radioligand with *K*_D_ ∼ 3 nmol/L in membranes from hmGlu2 receptor transfected CHO cells and rat cortex membranes (Te Riele et al. 2014; manuscript in preparation). Assay mixtures were incubated for 60 min at room temperature in 0.5 mL containing 75 *μ*g (hmGlu2 CHO or cortex) membrane protein. Nonspecific binding (about 30% of total binding) was estimated in the presence of 10 *μ*mol/L of JNJ-42341806. Filtration was performed using Unifilter-96 GF/C filters presoaked in 0.1% PEI and a 40-well manifold or 96-well Brandell harvester 96. After the addition of scintillation liquid, radioactivity on the filters was measured in a Microplate Scintillation and Luminescence Counter or Liquid Scintillation Analyzer (Perkin Elmer). Inhibition curves plotting percentage of total binding versus log concentrations of JNJ-40411813 were generated using GraphPad Prism (version 4.02) and analyzed using nonlinear regression analysis.

#### Selectivity assays

##### mGlu receptor panel

Ca^2+^ assays with human mGlu1, 3, 5, 7, or 8 receptor-expressing HEK 293 cells were performed as reported in Lavreysen et al. (2013), except for a slight change in the procedure for mGlu5: cells expressing the human mGlu5 receptor were seeded at 40,000 cells/well in MW384. Twenty-four hours after seeding, cells were incubated for 90 min in Ca^2+^ assay kit (Molecular Devices) dissolved in saline PBS supplemented with 5 mmol/L probenecid, pH 7.4 (f.c. 2.5 mmol/L probenecid as loading buffer was added on the cell layer without removal of medium) before measurements.

Measurement of [^35^S]GTP*γ*S binding to membranes from CHO cells expressing the rat mGlu6 receptor and membranes from L929sA cells expressing the human mGlu4 receptor were conducted as described in Lavreysen et al. 2013.

##### Additional selectivity assays

The effect of JNJ-40411813 on [^3^H]ketanserin binding to human 5HT_2A_ receptors expressed in NIH3T3 cells was evaluated; JNJ-40411813 was further assessed for activity at the D_2_ receptor using the [^35^S]GTP*γ*S assays performed in CHO cells stably expressing the human D_2L_ receptor. Membranes containing CHO cells and JNJ-40411813 were diluted in assay buffer containing 50 mmol/L Tris, pH 7.4, 100 mmol/L NaCl, 1 mmol/L ethylene glycol tetraacetic acid (EGTA), 3 mmol/L MgCl_2_, 10 *μ*mol/L GDP, 10 *μ*mol/L Dithiothreitol (DTT). The assay buffer used for diluting the membranes additionally contained 10 *μ*g/mL saponin. The procedure followed for the [^35^S]GTP*γ*S assay was similar to that used for CHO cells expressing the hmGlu2 receptor. [^35^S]GTP*γ*S was used at a final concentration of 0.25 nmol/L, and 10 *μ*mol/L dopamine was used as a positive control.

JNJ-40411813 was also tested at a concentration of 10 *μ*mol/L by CEREP (Celle L'Evescault, France) for its inhibition of radioligand binding to a battery of neurotransmitter and peptide receptors, ion channels, and transporters. Functional 5HT_2A_ receptor assays were also performed at CEREP. These studies were conducted as per standard protocols.

### In vivo pharmacology

#### mGlu2 and 5HT_2A_ receptor occupancy

Ex vivo mGlu2 occupancy studies were performed using [^3^H]JNJ-46281222 (Te Riele et al. 2014; manuscript in preparation). A dose–response experiment was performed to measure the ED_50_ (dose at which there is 50% receptor occupancy) for mGlu2 occupancy 1 h following JNJ-40411813 (p.o.) administration. After sacrifice, rat brains were immediately removed from the skull and rapidly frozen. The mGlu2 occupancy was measured in the striatum from individual rats as follows: brain sections (20 *μ*m thick) were incubated for 10 min with 1 nmol/L [^3^H] JNJ-46281222 in 50 mmol/L Tris-HCl, pH 7.4 containing 2 mmol/L MgCl_2_, 2 mmol/L CaCl_2_, and 0.3% Bovine serum albumin (BSA). The sections were washed and dried under a stream of cold air and exposed in a *β*-imager-2000 (Biospace Lab, Paris, France) for 4 h. Radioactivity from striatal tissue was quantified using the Beta Vision+ program (Biospace Lab). Nonspecific binding was measured on adjacent sections in presence of 10 *μ*mol/L JNJ-42341806. Specific binding was calculated as the difference between total binding and nonspecific binding. Percentage of receptor occupancy by the drug corresponded to 100% minus the percentage of receptor labeling. For the determination of ED_50_ values, the percentage of receptor occupancy was plotted against dosage and the sigmoidal log dose–effect curve of best fit was calculated by nonlinear regression analysis using the GraphPad Prism program (version 4.02).

A time-course occupancy experiment was performed after s.c. (2.5 and 10 mg/kg) and p.o. (5 and 20 mg/kg) administration of JNJ-40411813 in male Wistar rats. The animals were sacrificed at specific time points (0.5, 1, 2, 4, 8, and 24 h after drug administration). Brains were processed as described above. To enable PK-PD modeling, levels of JNJ-40411813 were also analyzed in brain and plasma of each individual rat (PK analysis section). Data were analyzed using a relative maximal effect (*E*_max_) model.

To evaluate 5HT_2A_ receptor occupancy, male Sprague–Dawley rats were treated with vehicle or increasing doses of JNJ-40411813 (s.c. or p.o.); the 5HT_2A_ receptor radioligand [^3^H]MDL-100907 (10 *μ*Ci/animal) was injected intravenously (i.v.) 30 min before sacrifice. Brains were immediately dissected from the skull and rapidly frozen in dry ice-cooled 2-methylbutane (−40°C). Sections (20 *μ*m thickness) were cut using a Leica CM 3050S cryostat-microtome (Leica, Brussels, Belgium), and thaw-mounted on microscope slides (SuperFrost Plus, LaboNord, France). Three frontal cortical sections and one cerebellum section were collected per glass slide. Brain sections were loaded in a *β*-imager-2000 (Biospace Lab) for 8 h and radioactivity emerging from the delineated brain area was quantified using the Beta Vision + program (Biospace Lab). The specific binding was calculated as the difference between the total binding in the frontal cortex and the cerebellum. The specific binding of [^3^H]MDL-100907 in frontal cortex of drug-treated rats was expressed as the percentage of specific binding in vehicle-treated rats. Percentage occupancy of the drug corresponded to 100% minus the percentage labeling in the treated animal. For the determination of ED_50_ values, the percentage of receptor occupancy was plotted against dose and the sigmoidal log dose–effect curve of best fit was calculated by nonlinear regression analysis using the GraphPad Prism software (version 4.02).

#### Sleep–wake architecture in rats

The effect of oral administration of JNJ-40411813 on sleep–wake organization in rats was performed as described earlier (Ahnaou et al. 2009). In brief, male Sprague–Dawley rats were surgically implanted with electrodes for recording the cortical electroencephalogram (EEG), electrooculogram (EOG) and electromyogram (EMG). Recordings were performed for 20 h after p.o. administration of saline (*n* = 32) at the acrophase of sleep, followed by recordings for the same duration after acute p.o. administration of JNJ-40411813 (3, 10, and 30 mg/kg) and of vehicle (control) (*n* = 8 for each condition). Sleep–wake states and related variables were analyzed over 20 continuous hours using discriminative analysis based on 5 EEG frequency domain values (*δ*: 0.5–4 Hz, θ: 4.2–8 Hz, *α*: 8.2–12 Hz, *σ*: 12.2–14 Hz, *β*: 14.2–30 Hz, *γ*: 30.2–50 Hz), integrated EMG, EOG, and body activity level. Different sleep–wake parameters were calculated in each hour for the amount of time spent in each vigilance state, number and duration of sleep period, onset latencies, and the number of transitions between states.

### Pharmacokinetic analysis

Male Sprague–Dawley rats were administered a single i.v. (2.5 mg/kg; i.v. arm) or p.o. (2.5, 5, 10, or 20 mg/kg; p.o. arm) dose of JNJ-40411813. All rats had ad libitum access to food and water prior to study drug administration and throughout the study. Blood was collected (via tail vein) at 0.11, 0.33, 1, 2, 4, 7, and 24 h post dose for the i.v. arm, and at 0.5, 1, 2, 4, 7, and 24 h post dose for the p.o. arm, centrifuged; plasma obtained was processed for protein precipitation and subsequently analyzed for JNJ-40411813 using a liquid chromatography/tandem mass spectrometry (API 4000 ™ LC/MS/MS system, Applied Biosystems, Cheshire, UK).

Similarly, brain and plasma levels of JNJ-40411813 were analyzed in a time-course mGlu2 occupancy study and correlated with the percent occupancy of JNJ-40411813 in the same individual animal. An additional step of homogenization of brain tissue in nine parts distilled water was performed.

### In vitro and in vivo metabolism

#### In vitro metabolism and identification

For metabolite identification purposes, JNJ-40411813 was incubated with human, rat, or mouse liver microsomes for 60 min at a substrate concentration of 5 *μ*mol/L; and with human hepatocytes for 60 min at substrate concentrations of 5 and 50 *μ*mol/L. Control incubations involved quenching with DMSO immediately after the addition of compound. Data were acquired on a Waters Acquity UPLC coupled with a QToF Premier mass spectrometer (Waters Corporation, Milford MA).

In combination with the recombinant CYP identification study (data not shown), inspection of the data suggested that four of the metabolites were accessible for NMR characterization. Scaled up incubations at 50 mmol/L in recombinant human (rh) CYP2D6 and 100 mmol/L in rhCYP3A4 were extracted and submitted for metabolite isolation and analysis by NMR.

#### In vivo excretion pathways

Rats were dosed with JNJ-40411813 (10 mg/kg, p.o.), urine samples were collected over 3 separate periods (0–2, 2–7, and 7–24 h post dose) and analyzed for drug-related material by LC/MS. For data interpretation, the terms major and minor are relative to each other, based on ion currents and should not be used as a formal quantification measure.

### Statistics

In rats, value of vigilance states expressed as means ± SEM were analyzed by a mixed-model anaylsis of variance (ANOVA) to assess the influence of JNJ-40411813 on vigilance states and related variables. For all analyses, the significance level was set at *P* < 0.05.

## Results

### In vitro

#### Functional effects of JNJ-40411813 in recombinant cell lines

**Figure 2:**
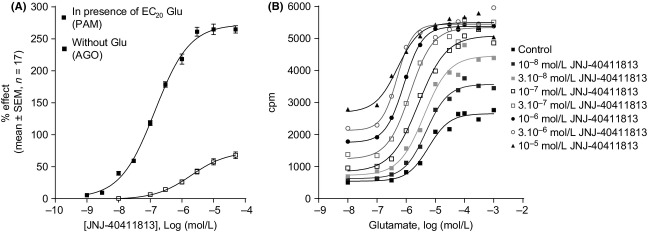
Effect of JNJ-40411813 on glutamate-induced [^35^S]GTP*γ*S binding at the mGlu2 receptor. (A) [^35^S]GTP*γ*S concentration–response curve of JNJ-40411813 in the absence or presence of an EC_20_ glutamate concentration. Data are expressed as percentage of the maximal response to glutamate and are mean ± SEM of 17 experiments. (B) Glutamate-induced [^35^S]GTP*γ*S binding in the absence or presence of increasing concentrations of JNJ-40411813. Data are from 1 representative experiment. The experiment was repeated with similar results. PAM, positive allosteric modulator; AGO, agonist; Glu, glutamate; CPM, counts per minute.

JNJ-40411813 demonstrated PAM activity at the hmGlu2 receptor by potentiating the glutamate (4 *μ*mol/L)-induced [^35^S]GTP*γ*S binding up to 273 ± 32%, with an EC_50_ of 147 ± 42 nmol/L (*n* = 17, Fig.2A). JNJ-40411813 also demonstrated agonist activity with an EC_50_ of 2159 ± 1069 nmol/L and maximal response of 74 ± 26% (*n* = 17, Fig.2A). The potency of glutamate increased upon addition of increasing concentrations of JNJ-40411813; the EC_50_ of glutamate decreased from 5.8 to 0.5 *μ*mol/L with the addition of 10 *μ*mol/L JNJ-40411813 (Fig.2B).

The effect of JNJ-40411813 was also confirmed on the rat mGlu2 receptor as it potentiated the effect of glutamate with an EC_50_ of 370 ± 120 nmol/L (Eff_curve_ was 479 ± 33%; *n* = 4). In stably transfected HEK293 cells expressing the hmGlu2 receptor, JNJ-40411813 potentiated glutamate-induced Ca^2+^ signaling with an EC_50_ of 64 ± 29 nmol/L (*n* = 33). JNJ-40411813 did not demonstrate mGlu2 receptor agonist activity up to 100 nmol/L (EC_50_ was 1843 ± 905 nmol/L; *n* = 25).

**Figure 3:**
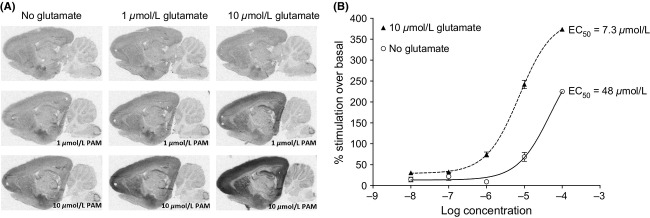
[^35^S]GTP*γ*S autoradiography on rat brain tissue. (A) Autoradiograms of sagittal rat brain sections showing [^35^S]GTP*γ*S binding in the absence and presence of glutamate (10 *μ*mol/L) and/or JNJ-40411813 (10 *μ*mol/L). Shown is one representative experiment (one individual rat). (B) [^35^S]GTP*γ*S concentration–response curve of JNJ-40411813 quantified in cortex in absence and presence of 10 *μ*mol/L glutamate.

**Figure 4:**
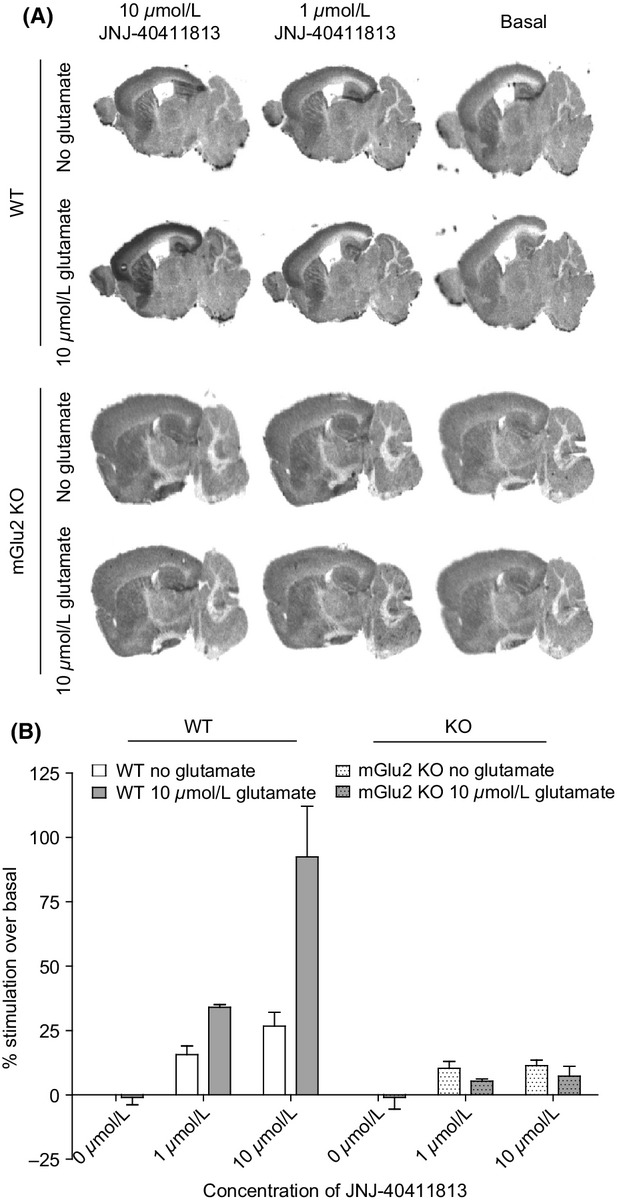
[^35^S]GTP*γ*S stimulation by JNJ-40411813 in mGlu2 wild-type (WT) and knockout KO mice. (A) Sagittal mice brain sections incubated with various concentrations of JNJ-40411813 in absence and presence of 10 *μ*mol/L glutamate. (B) Plot of [^35^S]GTP*γ*S binding versus concentration of JNJ-40411813.

Although application of 10 *μ*mol/L glutamate alone did not increase the [^35^S]GTP*γ*S signal significantly (0–5% over basal), coaddition of JNJ–40411813 clearly potentiated its response in brain regions known to express the mGlu2 receptor, more specifically in the cortical regions, striatum, and the hippocampus (Fig.3A). In the cortex, JNJ-40411813 alone stimulated basal binding with an EC_50_ of 48 *μ*mol/L and a maximal effect of ∼220%. When combined with 10 *μ*mol/L glutamate, JNJ-40411813 stimulated [^35^S]GTP*γ*S binding with an EC_50_ of 7.3 *μ*mol/L and a maximal effect of ∼380% (Fig.3B). These effects were absent in mGlu2 KO mice (Fig.4A and B), indicating that they are exclusively mGlu2 receptor mediated.

#### Radioligand binding to hmGlu2 CHO membranes or rat cortical membranes

**Figure 5:**
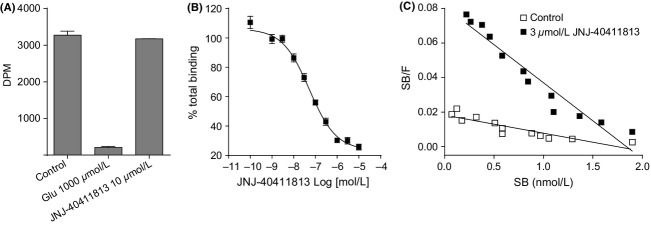
Binding of JNJ-40411813 to orthosteric versus allosteric mGlu2 binding site. (A) Inhibition of [^3^H]LY341495 binding to the hmGlu2 receptor, by glutamate and JNJ-40411813. Data are from a representative experiment (mean ± SD of duplicate determinations in one experiment). The experiment was repeated with similar results. Glu, glutamate. (B) Inhibition of [^3^H]JNJ-40068782 binding to hmGlu2 by JNJ-40411813. Data points represent mean percentage of total binding ± SEM. (11 experiments). (C) [^3^H]DCG-IV Scatchard Plot in the absence and presence of 3 *μ*mol/L of JNJ-40411813 (binding performed on membranes from hmGlu2 CHO cells).

JNJ-40411813 at 10 *μ*mol/L did not displace binding of the orthosteric mGlu2 receptor antagonist [^3^H]LY341495, indicating that JNJ-40411813 does not bind to the orthosteric receptor site (Fig.5A). JNJ-40411813 displaced binding of [^3^H]JNJ-40068782, a PAM acting on the mGlu2 receptor, with an IC_50_ of 68 ± 29 nmol/L (*n* = 11) in CHO cells (Fig.5B) and 83 ± 28 nmol/L (*n* = 2; data not shown) in membranes prepared from rat cortex. Binding inhibition was also confirmed using [^3^H]JNJ-46281222, a novel radioligand that allowed the evaluation of ex vivo occupancy after systemic administration of JNJ-40411813. A summary of EC_50_ and *K*_i_ values is shown in Table1.

**Table 1:** Summary of in vitro [^35^S]GTP*γ*S and mGlu2 radioligand binding data

	[^35^S]GTP*γ*S		[^3^H]JNJ-40068782	[^3^H]JNJ-46281222
	EC_50_ (nmol/L)	%Eff_curve_	*K*_i_ (nmol/L)	*K*_i_ (nmol/L)
Human mGlu2 CHO	147 ± 42	273 ± 32	37 ± 16	86 ± 3
Rat mGlu2 CHO	370 ± 120	479 ± 33	–	–
Rat cortex	–	–	41 ± 14	51 ± 5

[^35^S]GTP*γ*S values were measured in the presence of an EC_20_ concentration of glutamate, whereas radioligand binding was performed in the absence of glutamate. EC_50_ is defined as the concentration producing half-maximal effect; %Eff_curve_ is the maximal response of the compound based on the fitted concentration–response curve and *K*_i_ is the apparent equilibrium inhibition constant; all values are mean ± SD derived from at least four independent [^35^S]GTP*γ*S experiments or two independent radioligand binding experiments.

#### [^3^H]DCG-IV binding

The saturation curve of [^3^H]DCG-IV, an orthosteric mGlu2/3 agonist, was shifted to left in the presence of 3 *μ*mol/L of JNJ-40411813, with *K*_D_ values of 200 nmol/L in the absence of JNJ-40411813 and 30 nmol/L in the presence of JNJ-40411813 (Fig.5C). The *B*_max_ values of DCG-IV were similar in presence (20,050 fmol/mg) and absence (22,710 fmol/mg) of JNJ-40411813.

#### mGlu receptor selectivity

JNJ-40411813 did not activate any of the human mGlu receptor subtypes or the rat mGlu6 receptor up to 10 *μ*mol/L. Similarly, JNJ-40411813 up to 10 *μ*mol/L did not inhibit glutamate-induced signaling at any of the receptors, except in human mGlu5 and mGlu7 receptor-expressing cells; pIC_50_ values of 5.15 and 5.33, respectively, indicated minor antagonist effects at these receptors. Importantly, JNJ-40411813 had negligible PAM activity at the mGlu3 receptor (EC_50_ 11 *μ*mol/L), indicating that it selectively modulates mGlu2 function (Table2).

**Table 2:** In vitro functional effects of JNJ-40411813 on mGlu receptors

	IC_50_/EC_50_ (*E*_max_/Eff curve)			
Species					Receptor^1^	Antagonist	Agonist	PAM
Group I				
Human	mGlu1 (Ca^2+^)	>10 *μ*mol/L	>10 *μ*mol/L	n.d.
Human	mGlu5 (Ca^2+^)	7.17 *μ*mol/L (43%)	>10 *μ*mol/L	>30 *μ*mol/L
Group II				
Human	mGlu2 (GTP*γ*S)	–	2159 nmol/L (74%)	147 nmol/L (273%)
Human	mGlu2 (Ca^2+^)	–	1843 nmol/L	64 nmol/L
Human	mGlu3 (Ca^2+^)	>10 *μ*mol/L	>30 *μ*mol/L	11 *μ*mol/L
Group III				
Human	mGlu4 (GTP*γ*S)	>10 *μ*mol/L	>10 *μ*mol/L	n.d.
Rat	mGlu6 (GTP*γ*S)	>10 *μ*mol/L	>10 *μ*mol/L	n.d.
Human	mGlu7 (Ca^2+^)	4.68 *μ*mol/L (77%)	>10 *μ*mol/L	n.d.
Human	mGlu8 (Ca^2+^)	>10 *μ*mol/L	>10 *μ*mol/L	n.d.

n.d., not determined.

1 The pharmacological read-out used to detect each activity is indicated in brackets.

#### Broad receptor profiling

JNJ-40411813 demonstrated weak affinity for the human 5HT_2C_, adrenergic *α*2A, *α*2B, and rat Na^+^ channel and moderate affinity for the human 5HT_2A_ receptor (IC_50_ = 708 nmol/L; Table3). JNJ-40411813 up to 30 *μ*mol/L did not activate the D_2_ receptor (data not shown).

**Table 3:** In vitro binding affinities of JNJ-40411813 for various receptor binding sites and ion channels

Target	pIC_50_
Receptor	
h5HT_2A_	6.15
h5HT_2B_	<5
h5HT_2C_	∼5.2
h5HT_1A_	<5
h5HT_1B_	<5
h5HT_1D_	<5
h5HT_4b_	<5
h5HT_7_	<5
hAa1A	<5
hAa2A	5.12
hAa2B	5.33
hAa2C	<5
hD2L	<5
hH1	<5
Ion channels	
rCa^2+^ channels	<5
rNa^+^ channel	5.75
hERG	<5

Additional studies conducted at CEREP indicated that JNJ-40411813 (up to 10 *μ*mol/L) inhibited binding at the 5HT_2A_ receptor (93% inhibition), but did not produce a significant (>50%) binding inhibition of any of the other targets investigated. JNJ-40411813 inhibited serotonin-induced Ca^2+^ signaling at the human 5HT_2A_ receptor, with a *K*_b_ value of 1.1 *μ*mol/L and *E*_max_ of 70%, indicating that it acts as a weak 5HT_2A_ antagonist.

### In vivo

#### mGlu2 and 5HT_2A_ receptor occupancy

**Figure 6:**
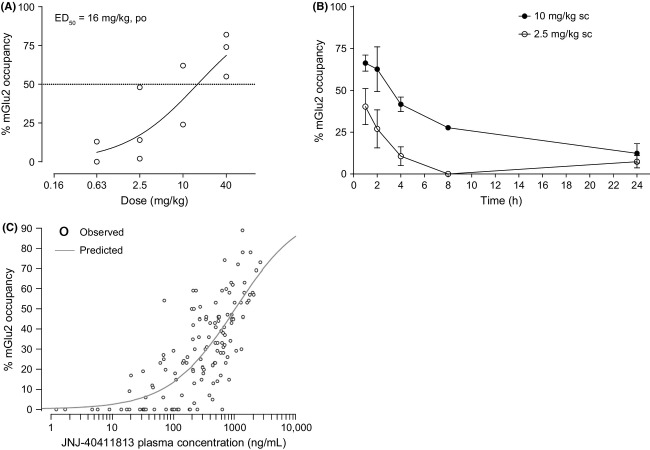
Ex vivo mGlu2 receptor occupancy of JNJ-40411813 in male Wistar rats. (A) mGlu2 receptor occupancy in function of JNJ-40411813 dose (1 h after p.o. dosing; three rats per dose). (B) Time-course of ex vivo mGlu2 receptor occupancy after s.c. administration (2.5 and 10 mg/kg) of JNJ-40411813 in rats (for each dose, three rats per time point). (C) Occupancy–plasma level relationship of JNJ-40411813_._ JNJ-40411813 plasma concentrations were analyzed in each rat used in the occupancy time-course experiments (for each dose route and dose level, *n* = 3 per time point). Each data point represents a single animal.

**Figure 7:**
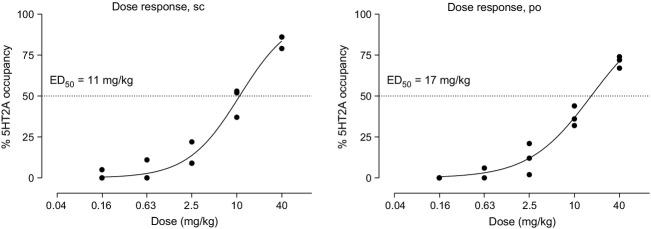
In vivo 5HT_2A_ receptor occupancy of JNJ-40411813 in male Sprague–Dawley rats. Plot of percentage of 5HT_2A_ receptor occupancy versus dose of JNJ-40411813, after single s.c. or p.o. administration at different dose levels.

In a dose–response experiment, JNJ-40411813 exhibited mGlu2 receptor occupancy with an ED_50_ of 16 mg/kg 1 h after p.o. dosing (Fig.6A). In a time-course occupancy experiment, JNJ-40411813 demonstrated maximum occupancy of rat brain mGlu2 receptors 0.5–1 h after s.c. (10 mg/kg) or p.o. (20 mg/kg) dosing. A decrease in mGlu2 receptor occupancy was observed over time, with about 50% occupancy detected up to 4 h after s.c. (10 mg/kg) dosing (Fig.6B). Dosing at different routes and time points allowed refined PK-PD modeling; since plasma–occupancy relationships obtained after s.c. and p.o. dosing were overlapping (data not shown), data from both treatment groups were pooled to give a better estimation of the PK/PD parameters. Based on the collective plasma–occupancy relationship, the estimated concentration observed at 50% of the maximum response was 1032 ng/mL for plasma (Fig.6C). JNJ-40411813 occupied the 5HT_2A_ receptor with an ED_50_ of 11 mg/kg after s.c. and 17 mg/kg after oral p.o. administration (Fig.7).

#### Sleep–wake architecture in rats

**Figure 8:**
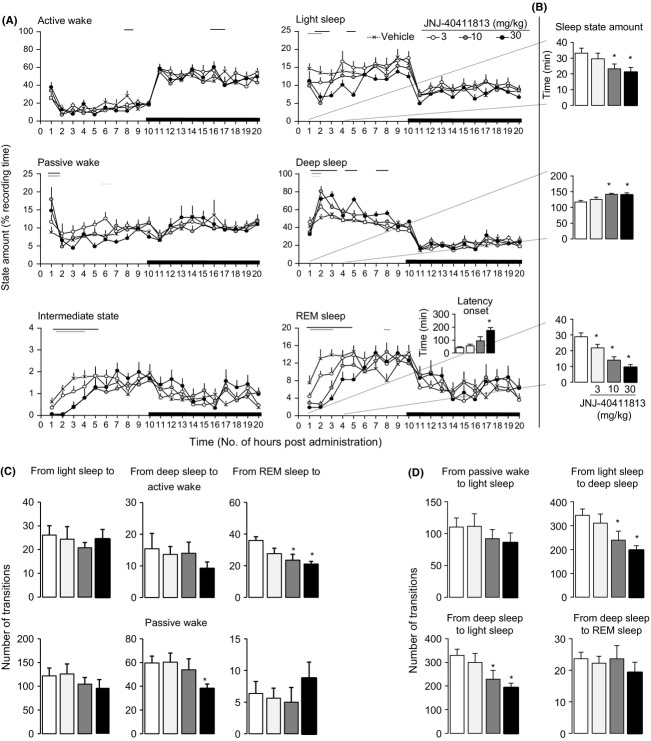
Effects of oral administration of JNJ-40411813 (3, 10, and 30 mg/kg) and of vehicle (20% HPCD+1HCl) on sleep–wake organization. (A) Sleep–wake architecture in rats during 20 consecutive hours of the recording session, and small bottom right panel indicates the latency to REM sleep onset. (B) Amount of time spent in light sleep, deep sleep, and REM sleep during the first 4 h of the recording session. Mean values of eight rats expressed as percentages of the recording time for the states active wake, passive wake, intermediate stage, light sleep, deep sleep, and REM sleep. Open and dark areas in the abscissa axis indicate light and dark phase of the circadian time, respectively. Horizontal color coded lines under curves indicate statistically significant difference between vehicle and drug-dose groups. (C) State transitions from sleep states toward active and passive waking. (D) Progression state transition from waking to sleep states. Mean values of eight rats, expressed in minutes for vigilance states and number of transitions. *P < 0.05 indicate statistically significant difference versus control (mixed ANOVA) between vehicle and drug-dose groups.

Acute p.o. administration of JNJ-40411813 (3, 10, and 30 mg/kg) elicited consistent changes in the distribution of sleep–wake states, and notably decreased the total amount of rapid eye movement (REM) sleep (treatment × time interaction, *F*[5, 1172] = 2.1789, *P* < 0.0001) and increased time spent in deep sleep (treatment × time interaction, *F*[5, 1172] = 2.076, *P* < 0.0001) (Fig.8A).

When restricted to the first 4 h of the recording session (Fig.8B), the mixed-model ANOVA revealed that JNJ-40411813 dose dependently reduced the time spent in REM sleep (treatment × time interaction, *F*[9, 212] = 2.16, *P* < 0.05) and light sleep (treatment × time interaction, *F*[9, 212] = 3.44, *P* < 0.005), while it increased the time spent in deep sleep relative to control (treatment × time interaction, *F*[9, 212] = 3.45, *P* < 0.005). A lowest active dose (LAD) of 3 mg/kg p.o. was noted.

A detailed analysis of sleep variables showed that the decreased time spent in light sleep and REM sleep was derived from the decrease in the number of episodes and the mean duration of these vigilance states. The enhanced time spent in deep sleep resulted from an increase in the mean duration, whereas the number of periods of this vigilance state remained unchanged (data not shown). Examination of total number of state transitions indicated that JNJ-40411813 consistently reduced switches from deep sleep to passive waking (“treatment × time” interaction: *F*[9,212] = 2.6, *P* < 0.05), and from REM sleep to active waking (“treatment” *F*[3, 28] = 4.9, *P* < 0.05), “time” *F*[3, 212] = 0.9, *P* = 0.46); however, the ANOVA interaction was not significant (“treatment × time”; *F*[9, 212] = 1.3, *P* = 0.19) (Fig.8C). The potent enhancing effect on deep sleep duration was consistent with marked reduction of transitions from light sleep to deep sleep stages (“treatment × time” interaction: *F*[9, 212] = 3.2, *P* < 0.005) suggesting a direct effect of JNJ-40411813 on the mechanism of initiation and maintenance of stable deep sleep episodes (Fig.8D). Concomitant to changes in REM sleep state, JNJ-40411813 consistently lengthened the REM sleep onset latency (Fig.8A right bottom upper panel).

### Pharmacokinetics of JNJ-40411813

**Figure 9:**
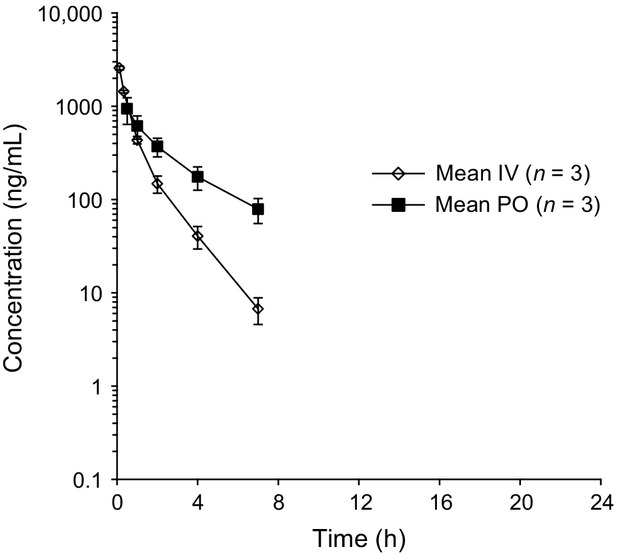
Plasma concentrations after single oral administration of 10 mg/kg JNJ-40411813 and single i.v. administration of 2.5 mg/kg in the male Sprague–Dawley rat.

The PK analysis revealed that JNJ-40411813, after a single i.v dose of 2.5 mg, demonstrated a mean plasma exposure (AUC_0-∞_) of 1833 ± 90 ng.h/mL. The mean plasma clearance (CL) was 1.4 ± 0.1 L/h/kg, and the mean volume of distribution (*V*_dz_) was 2.3 ± 0.2 L/kg indicating distribution of the compound to other tissues. Plasma levels were below the limit of quantification at the 24-h time point (Fig.9). JNJ-40411813 was rapidly absorbed following a single p.o. administration to fed rats at a dose of 10 mg/kg, with a *C*_max_ of 938 ng/mL at 0.5 h post dose. The mean exposure (AUC_0-∞_) was 2250 ± 417 ng.hour/mL, resulting in an absolute oral bioavailability of 31%. Plasma levels decreased with a mean half-life (*t*_1/2_ [2–7 h]) of 2.3 ± 0.5 h.

Mean plasma concentration–time profiles were comparable after single p.o. dosing at 2.5, 5, 10, and 20 mg/kg, and *C*_max_ and AUC_0-∞_ values increased proportionally across the dose range tested (data not shown).

### Metabolism of JNJ-40411813

**Figure 10:**
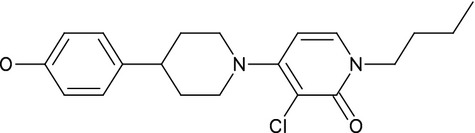
Metabolite JNJ-42159052 Identified by NMR.

JNJ-40411813 forms a variety of oxidative metabolites in human, rat, and mouse liver microsomes. The major metabolites excreted in rat urine were consistent with the in vitro findings. One of the JNJ-40411813 metabolites (JNJ-42159052; Fig.10) displayed relatively high 5HT_2A_ receptor affinity: JNJ-42159052 showed an IC_50_ of 102 nmol/L for blocking [^3^H]ketanserin binding to human 5HT_2A_ receptors.

## Discussion

Modulation of mGlu2/3 receptor activity has emerged as a novel potential therapeutic strategy for treating psychiatric and neurological disorders (Niswender and Conn 2010). Extensive research conducted on either direct agonist or allosteric modulator approaches to regulate mGlu2 receptor function is aimed to provide benefit in the treatment of these disorders (Trabanco et al. 2011; Trabanco and Cid 2013).

We showed that JNJ-40411813 exhibits mGlu2 PAM activity, both at cloned mGlu2 receptors as well as native receptors in the brain tissue. JNJ-40411813 at higher concentrations also induced modest activation of [^35^S]GTP*γ*S binding in absence of glutamate, suggesting that the compound has some intrinsic agonist efficacy. However, the possibility that this is, in fact, a modulation of endogenous, albeit low levels of glutamate cannot be excluded. JNJ-40411813 also increased the glutamate-induced [^35^S]GTP*γ*S signal in brain regions known to express the mGlu2 receptor (specifically in cortical regions, striatum, and hippocampus).

Unlike agonists that bind directly to the orthosteric binding site of the receptor, PAMs exhibit a unique mechanism of action wherein they bind to an allosteric binding site that is distant from the agonist binding site (Niswender and Conn 2010). JNJ-40411813 displaced [^3^H]JNJ-40068782 and [^3^H]JNJ-46281222, both PAMs of the mGlu2 receptor, but failed to displace [^3^H]LY341495, a competitive mGlu2/3 receptor antagonist, thus, confirming that it binds to an allosteric site. Furthermore, in presence of JNJ-40411813, the binding affinity of the mGlu2/3 receptor agonist DCG-IV was increased, without affecting the number of binding sites, thus confirming earlier observations (Lavreysen et al. 2013).

JNJ-40411813 demonstrated high selectivity for the mGlu2 receptor with no agonist, and antagonist activity for other mGlu receptors. Additionally, we did not find appreciable mGlu3 PAM activity (EC_50_ for mGlu3 PAM activity >150-fold EC_50_ for mGlu2 PAM activity), indicating that JNJ-40411813 acts as a selective mGlu2 PAM. JNJ-40411813 inhibited 5HT_2A_ receptor function in vitro, although at ∼10-fold higher concentrations compared with the mGlu2 receptor. JNJ-40411813 did not activate the D_2_ receptor (data not shown), indicating that JNJ-40411813 does not act via the dopaminergic system.

To our knowledge, this is the first time that direct rodent mGlu2 target engagement studies are reported. With use of the novel mGlu2-specific radioligand [^3^H] JNJ-46281222 (Te Riele et al. 2014; manuscript in preparation), we showed that JNJ-40411813 occupies the mGlu2 receptor after systemic administration, an important finding that permits the linking of receptor occupancy levels with efficacy in in vivo pharmacodynamic readouts or animal models of disease. PK-PD modeling resulted in an EC_50_ value of 1032 ng/mL.

Interestingly, although there seemed to be a good relationship between JNJ-40411813 plasma concentrations and receptor binding to the mGlu2 receptor, JNJ-40411813 unexpectedly also bound 5HT_2A_ receptors to the same level at similar doses, which could not be explained by the moderate in vitro 5HT_2A_ potency of JNJ-40411813. Further experiments revealed that JNJ-40411813 exhibited a longer duration of action for 5HT_2A_ versus mGlu2 receptor occupancy (data not shown). We found that in rats, JNJ-40411813 is metabolized to a variety of oxidative metabolites, including the metabolite JNJ-42159052, a relatively potent 5HT_2A_ receptor antagonist, which in all likelihood, contributes to and explains the relatively high 5HT_2A_ receptor binding in rats. These findings suggest that both mGlu2 receptor activity and 5HT_2A_ receptor antagonism can contribute to the preclinical in vivo activity profile of JNJ-40411813. Importantly, because this metabolite is not found to the same extent in humans, it is believed to be clinically irrelevant (Hoeben et al. 2013).

JNJ-40411813 significantly suppressed REM and promoted deep sleep in rats. Assessment of sleep–wake cycle is an accurate and validated tool that is sensitive to centrally active drugs. Glutamate and serotonin signaling are the key neurochemical components of sleep-promoting and arousal centers in the brain. Glutamate release shows rhythmic fluctuations reaching maximal levels during waking and REM sleep in cortical areas such as the rostromedial medulla, orbitofrontal, and prefrontal cortex (Kodama et al. 1998; Lopez-Rodriguez et al. 2007). The mGlu2 receptors are localized predominantly in presynaptic terminals of glutamate neurons, where they inhibit the release of glutamate. Therefore, it is not surprising that pharmacologic modulation of mGlu2 receptor would be effective in modulating vigilance states.

Previous pharmacological studies using mGlu2 receptor agonist and PAMs demonstrated that central or systemic activation of mGlu2 receptor consistently suppressed REM sleep and prolonged its onset latency in rats (Ahnaou et al. 2009; Fell et al. 2011; Siok et al. 2012; Lavreysen et al. 2013). The specific suppression effect on REM sleep was confirmed in wild type, but not in mGlu2 receptor knockout mice (Ahnaou et al. 2009). The concentration of JNJ-40411813 that achieved ≥30% mGlu2 receptor occupancy resulted in consistent inhibition of REM sleep, indicating that relatively low levels of occupancy may be sufficient for this particular effect.

The distribution of serotonin 5HT_2A_ receptors in the brain regions known to be important in the regulation of sleep and wake, such as the hypothalamus and thalamus, aligns with the growing body of preclinical and clinical data to support a role for 5HT_2A_ antagonists in promotion of deep sleep, and therefore these antagonists offer potential to address several unmet needs in insomnia pharmacotherapy (Vanover and Davis 2010; Zisapel 2012). It is likely that the functional effects of JNJ-40411813 on deep sleep observed in this study arise from an interaction with the 5HT_2A_ receptor via the metabolite JNJ-42159052. 5HT_2A_ antagonists were shown to have no significant effect on REM sleep (Morairty et al. 2008), further supporting the hypothesis that the observed reduced REM sleep is due to mGlu2 receptor activation.

The majority of hypothesized functions for deep sleep and REM sleep states, and in particular the one that received the most attention has been waking related. The idea that both sleep states are necessary for optimal cognitive function in waking has been the basis of a number of related hypotheses in the recent scientific literature. The dual-process hypothesis proposes that deep sleep is beneficial for declarative memories, whereas REM sleep is important for consolidation of nondeclarative, procedural, and emotional memories (Ackermann and Rasch 2014). The fact that REM sleep naturally follows deep sleep supports the complementing contributions of sequential deep sleep and REM sleep to synaptic form of consolidation for stabilizing memories. Accordingly, one would expect that suppression of REM sleep impairs memory consolidation. Pharmacological suppression of REM sleep in human by administration of antidepressants (selective noradrenaline or serotonin reuptake inhibitors) did not impair memory consolidation (Rasch et al. 2009), which is in agreement with clinical observations that antidepressant treatment does not affect memory function.

JNJ-40411813 when administered at a single dose of 10 mg/kg in fed rats was absorbed rapidly, with moderate oral bioavailability (31%). This could be attributed to low water solubility of JNJ-40411813, leading to incomplete in vivo dissolution and hepatic metabolism of the drug. JNJ-40411813 (2.5 mg/kg, i.v.) was well distributed to other tissues, due to both its lipophilic property and high cell permeability which enables easy permeability across the cell membrane. The mean plasma concentration–time profile of JNJ-40411813 was linear across the dose range tested.

In conclusion, JNJ-40411813, a novel, potent, and systemically active mGlu2 PAM, showed more than 50-fold selectivity for the mGlu2 receptor over other subtypes and moderate in vitro affinity for the 5HT_2A_ receptor. Nevertheless, it could not explain the equipotency observed in terms of mGlu2 and 5HT_2A_ occupancy. Rather, JNJ-40411813 metabolite accounted for additional 5HT_2A_ engagement in vivo. The combined mGlu2 PAM/5HT_2A_ antagonist profile of JNJ-40411813 was highlighted by an effect on REM sleep as well as deep sleep in rats. The in vivo behavioral effects of JNJ-40411813, which are described elsewhere (Lavreysen et al. submitted[Bibr b16]) prompted us to select JNJ-40411813 for further clinical development, which is ongoing.

## Abbreviations

ANOVA: anaylsis of variance
EEG: electroencephalogram
EMG: electromyogram
EOG: electrooculogram
HP-*β*-CD: hydroxypropyl-*β*-cyclodextrin
LAD: lowest active dose
NMDA: *N*-methyl-D-aspartate
REM: rapid eye movement
